# 202 Football Cooperative, a community-based physical activity social intervention for men: The development of an implementation strategy for scale-up

**DOI:** 10.1093/eurpub/ckae114.069

**Published:** 2024-09-26

**Authors:** Christopher Mcdermott

**Affiliations:** South East Technological University, Ireland

## Abstract

**Purpose:**

Gender-sensitised health promotion strategies are crucial for men for several reasons including; Addressing male-specific health issues, Improving mental health, and promoting healthy lifestyles. Football Cooperative (FC) provides social games aimed at enhancing overall health and well-being among men and has the potential to return benefits including decreased blood pressure, heart rate, and fat mass, which presents solutions to address existing challenges in men’s health.

This study aims to evaluate FC and gain insights into operating characteristics, both internally and externally, and learn from good practices, to develop a scalable implementation strategy for FC.

**Methods:**

The Consolidated Framework for Implementation Research (CFIR) guided qualitative data collection from various stakeholders for this study, including; Interviews (n = 26), Ad hoc Interviews (n = 9), and Focus Groups (n = 3). Ethnographic data was collected through researcher participation in weekly games and completing reflective logs (n = 8).

Data was recorded, transcribed verbatim, and analysed via thematic analysis. This analytical approach was guided by CFIR and in collaboration with a multi-disciplinary research team.

**Results:**

Six main determinants were identified from the data including 1: Culture defined by inclusivity and diversity, developing community, creating a pressure-free environment and embracing values such as respect, 2: Game Characteristics - which are the structure of how games are implemented, 3: Motivations to Participate - which are the key factors that bring men to FC games, 4: Benefits of Participation is concerned with the positive benefits the games have on their lives, 5: Facilitators of Participation defined by anything that makes games accessible to men, and 6: Barriers to Participation is defined by anything that make participation challenging.

**Conclusions:**

Various elements are essential to replicating FC at new sites and their inclusion should form a final strategy. Bringing the FC Culture to new venues, avoiding late start times, and ensuring flexibility when working with new stakeholders regionally are important, as are branding and communication alignment. Volunteer recruitment and management are at the core of FC and policy to support this is critical, as is the accessibility of games in terms of price, ability, and ease of joining games.

**Funding:**

South East Technological University & UEFA

